# Localized microglia dysregulation impairs central nervous system myelination in development

**DOI:** 10.1186/s40478-023-01543-8

**Published:** 2023-03-22

**Authors:** Rebecca K. Holloway, Liang Zhang, Irene Molina-Gonzalez, Kathy Ton, James A. R. Nicoll, James P. Boardman, Yan Liang, Anna Williams, Veronique E. Miron

**Affiliations:** 1 Keenan Research Centre for Biomedial Science at St. Michael’s Hospital, 209 Victoria Street, Toronto, ON M5B 1T8 Canada; 2 Department of Immunology, University of Toronto, Toronto, ON Canada; 3 United Kingdom Dementia Research Institute at The University of Edinburgh, Edinburgh, Scotland, UK; 4 Centre for Discovery Brain Sciences, Chancellor’s Building, The University of Edinburgh, Edinburgh, Scotland, UK; 5 Medical Research Council Centre for Reproductive Health, The Queen’s Medical Research Institute, The University of Edinburgh, Edinburgh, Scotland, UK; 6 Nanostring Technologies, Inc., Seattle, WA USA; 7 Clinical Neurosciences, Clinical and Experimental Sciences, Faculty of Medicine, University of Southampton, Southampton, UK; 8 Department of Cellular Pathology, University Hospital Southampton NHS Foundation Trust, Southampton, UK; 9 Centre for Regenerative Medicine, Institute for Regeneration and Repair, The University of Edinburgh, Edinburgh, Scotland, UK

**Keywords:** Myelin, Oligodendrocyte, White matter, Microglia, Neuroinflammation

## Abstract

Myelination of neuronal axons is a critical aspect of central nervous system development and function. However, the fundamental cellular and molecular mechanisms influencing human developmental myelination and its failure are not fully understood. Here, we used digital spatial transcriptomics of a rare bank of human developing white matter to uncover that a localized dysregulated innate immune response is associated with impeded myelination. We identified that poorly myelinating areas have a distinct signature of Type II interferon signalling in microglia/macrophages, relative to adjacent myelinating areas. This is associated with a surprising increase in mature oligodendrocytes, which fail to form myelin processes appropriately. We functionally link these findings by showing that conditioned media from interferon-stimulated microglia is sufficient to dysregulate myelin process formation by oligodendrocytes in culture. We identify the Type II interferon inducer, Osteopontin (SPP1), as being upregulated in poorly myelinating brains, indicating a potential biomarker. Our results reveal the importance of microglia-mature oligodendrocyte interaction and interferon signaling in regulating myelination of the developing human brain.

**Supplementary Information:**

The online version contains supplementary material available at 10.1186/s40478-023-01543-8.

## Introduction

Myelination of neuronal axons is required for optimal health and function of the central nervous system (CNS). This process initiates in early development and involves maturation of progenitors into oligodendrocytes, followed by the extension then wrapping of their myelin processes. Studies in rodent models have found that insult to the developing brain impairs myelination primarily via poor oligodendrocyte maturation [40]. Although CNS-resident macrophages, the microglia, have been implicated [13, 39], our recent work uncovered that they are not required for oligodendrocyte maturation nor developmental myelination [24]; in addition, microglia are not consistently activated across animal models of poor myelination [36]. Although microgliosis has been observed in human infant brains with poor myelination [12, 18, 42], the mechanisms by which these cells are involved are not fully understood, and unbiased comprehensive analysis is lacking. Therefore, the cellular and molecular mechanisms underpinning the failure of developmental myelination are still being uncovered.

Investigating developmental myelination specifically in human brain is important as there are critical differences to rodents with regards to timing of myelination and oligodendrocyte transcriptomes [14]. Nonetheless, human infant brain analyses have been hampered by limited tissue availability and inter- and intra-individual variability in the extent of myelination, thereby making it difficult to extract conserved mechanisms using traditional histological analyses. To overcome these issues, we hypothesized that performing transcriptomic analysis of human developing white matter using multiplexed spatial transcriptome profiling [31] on a rare bank of tissue would allow for localized comparison of normally versus poorly myelinating areas and infants, thereby uncovering the critical interactions and pathways involved in human myelination failure.

## Results

### Digital spatial transcriptomic profiling of human infant white matter

**Fig. 1:**
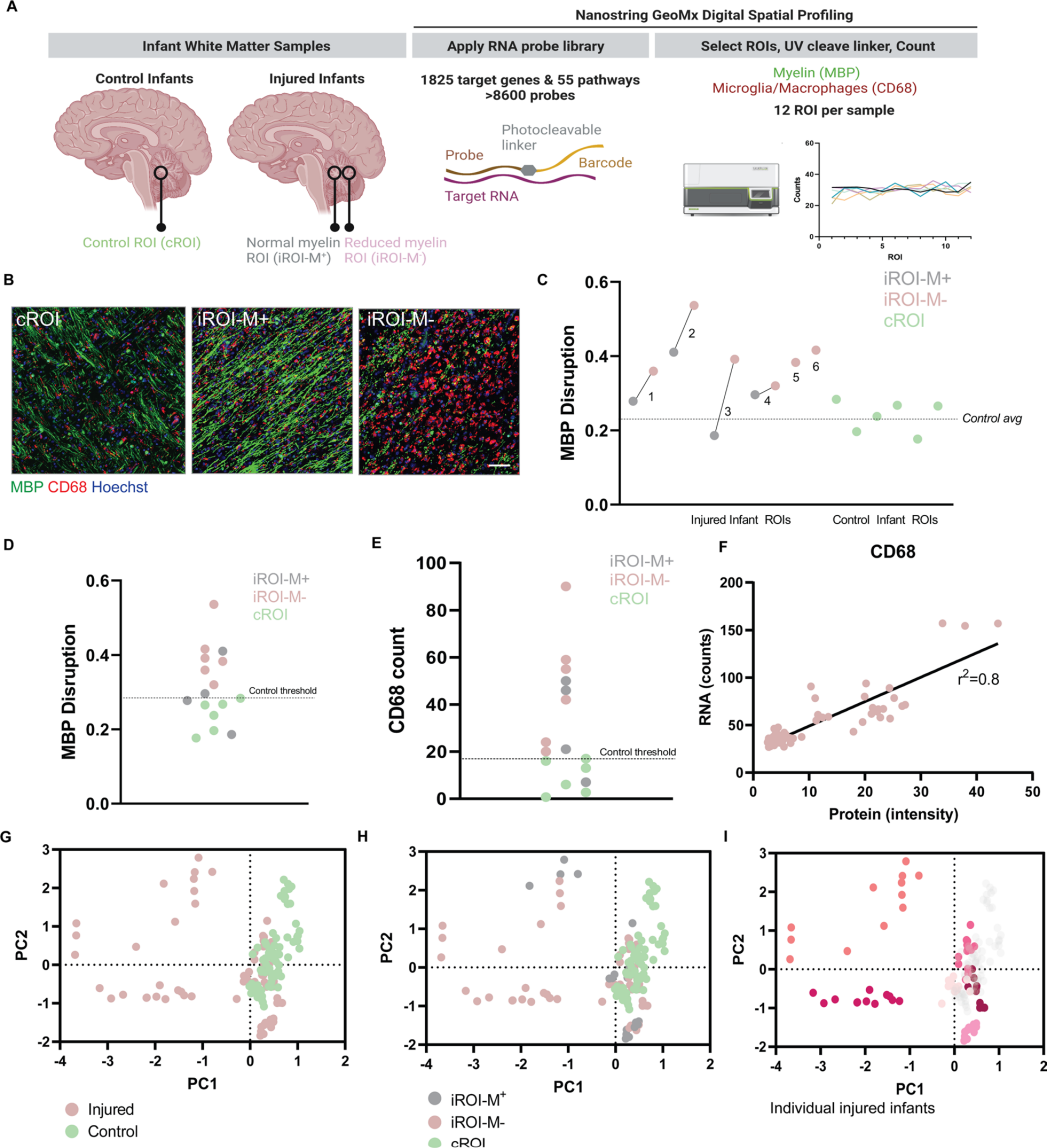
Digital spatial transcriptomic profiling of human developmental white matter.** A** Diagram of experimental approach. Regions of interest (ROI) were assessed in infant brain white matter samples (cerebellum), with control ROI (cROI) in control infants compared to ROI in injured infants (iROI) which were normally myelinating (iROI-M^+^) or showed reduced myelination (iROI-M^−^). A probe library targeting 1825 genes in 55 pathways was applied to the tissue, and 12 ROIs were selected per sample based on levels of myelin basic protein (MBP; green) and microglia/macrophages (CD68; red), counterstained with Hoechst (cyan). Probes were cleaved with UV light and RNA counts performed per ROI. **B** Example ROIs from cROI, iROI-M^+^, and iROI-M^−^ stained for MBP (green) and CD68 (red) and counterstained for Hoechst (blue). Scale bar, 65 µm. **C** MBP Disruption as measured by average MBP Sphericity per infant in injured iROI-M^+^ (grey), iROI-M^−^ (pink) and cROI (green). **D** MBP Disruption as measured by average MBP Sphericity in all infant ROIs, with highest level in control indicated as threshold (dotted line). **E** Average CD68 count in all infant ROIs, with highest level in control indicated as threshold (dotted line). **F** *CD68* RNA counts versus CD68 protein intensity measurements in respective injured samples. N = 6 infants, n = 71 ROI. Goodness of fit, r^2^ = 0.8. Slope is significantly non-zero, *P* < 0.0001. **G** Principal component analysis (PCA) plot of ROI from control infants (cROI; green) and injured infants (iROI; pink). **H** PCA plot of cROI (green), iROI-M^+^(grey), and iROI-M^−^ (pink). **I** PCA plot of all ROI showing individual injured cases in distinct colours

Analyses of human infant post-mortem CNS is extremely challenging due to limited availability worldwide. Nonetheless, we collected a rare bank of developing white matter of a small number of control and injured cases (Additional file 1: Table S1), albeit to our knowledge, the largest cohort for such analyses to date. We performed digital spatial transcriptomic profiling (DSP), assessing regions of interest (ROI) from control infants who died without brain injury (cROI; n = 6 infants) and injured infants with myelination failure (iROI; n = 6 infants) (Fig. 1A; Additional file 1: Table S1). The latter were classified by histology to have hypoxic-ischemic encephalopathy (HIE)/hypoxia or periventricular leukomalacia (PVL). ROI in injured infants, termed iROI, were divided into 2 categories (Fig. 1B–D). iROI with reduced and abnormal myelination were termed iROI-M^−^, characterized by a decreased amount of myelin protein and/or myelin disruption (myelin with a blebbed and distorted appearance, quantified as sphericity of stain). iROI with normal myelination as indicated by linear myelin sheaths were termed iROI-M^+^. ROI from control infants were termed cROI. Increased microglia/macrophage densities (CD68 + cells) were observed in iROI (Fig. 1E) consistent with known microgliosis in white matter of injured human infant brain [12, 40, 42]. Injured infants were sex-matched and closely age-matched to controls, with the exception of the one preterm injury case which could not be age-matched due to low gestational age at death (Additional file 1: Table S1). We selected developing white matter of the cerebellum due to its robust myelination in deep white matter in early human development which facilitated the delineation of poorly myelinating versus normally myelinating regions, and due to its vulnerability to injury [29, 34, 43] (Additional file 1: Fig. S1A). We used the Nanostring GeoMx platform which is compatible with archived paraffin-embedded sections, applying 8684 UV-cleavable barcoded RNA probes against 1825 genes encompassing 55 major pathways. Twelve ROI (650 µm × 650 µm) per infant were selected based on abovementioned myelin disruption and microglia/macrophage measurements, and approximately 6 million reads/ROI were detected across all samples with 822 targets detected above the negative probe signal:noise threshold. Probe counts were averaged per target then normalized against the 75th percentile signal from the respective ROI (‘Q3 normalization’; Additional file 2: Sheet 1). As a proof-of-concept, we confirmed that *CD68* RNA counts were highly correlated with respective CD68 protein intensity for all iROI (Fig. 1F). Principal component analysis revealed that iROI were less tightly clustered than cROI (Fig. 1G, H), likely reflecting variability and extent of injury. Some variability could be explained by sex, with females less tightly clustered than males, whereas clustering was not influenced by age or neuropathological description of type of injury (Additional file 1: Fig. S1B–D). The largest transcriptomic differences were seen between infants (Fig. 1I), as expected with injured human tissue based on adult human brain transcriptomic studies [14, 23, 33].

### Type II interferon signalling in microglia/macrophages is spatially associated with poor developmental myelination

**Fig. 2:**
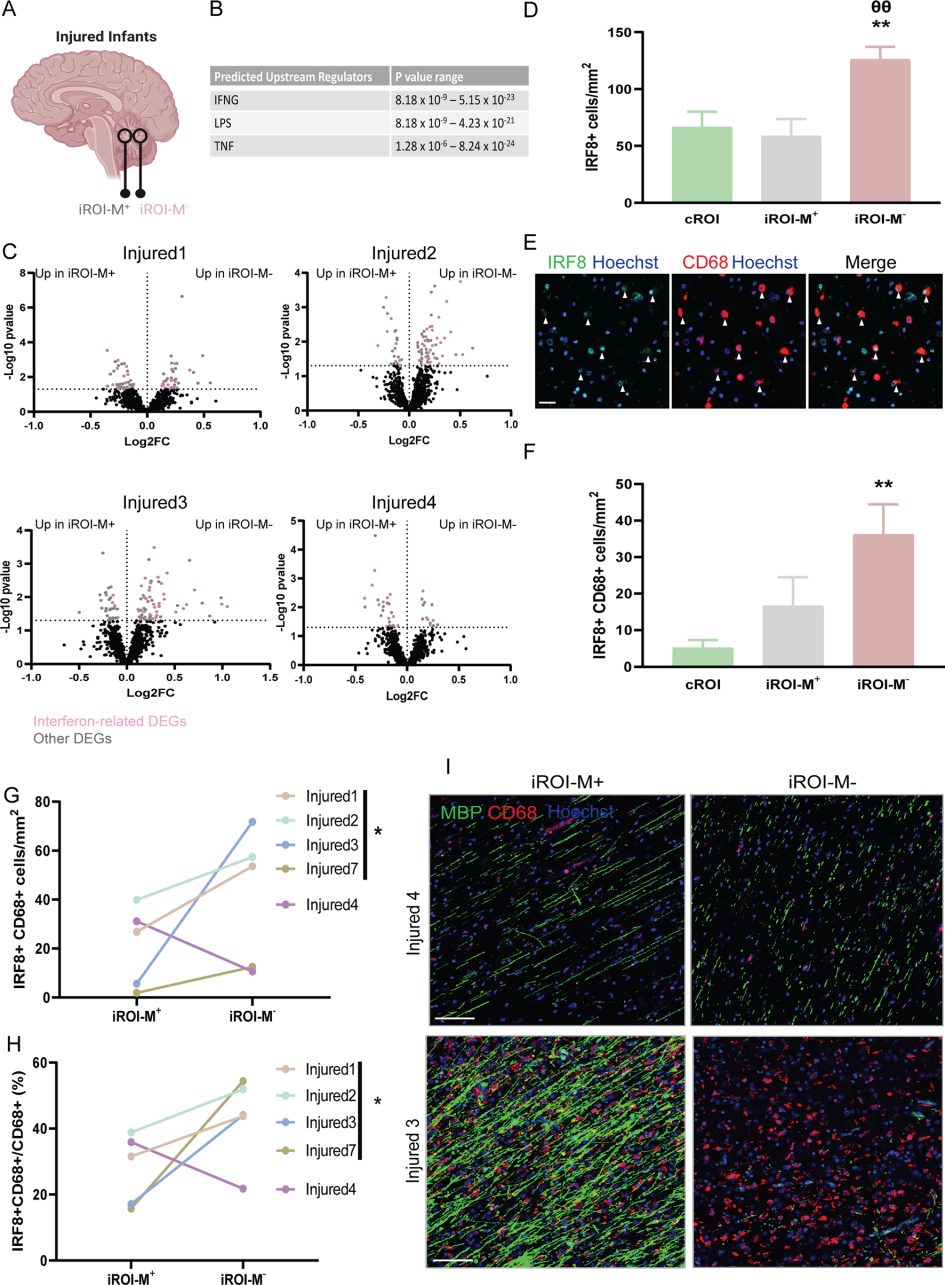
Type II interferon pathway engagement in microglia/macrophages in poorly myelinating regions of developing white matter. **A** Diagram of approach comparing iROI-M^+^ and iROI-M^−^ from respective samples. **B** Ingenuity Pathway Analysis (IPA) of genes differentially expressed between iROI-M^+^ and iROI-M^−^ indicated predicted upstream regulators IFNG, LPS, and TNF, which are part of the Type II interferon response. **C** Volcano plots of iROI-M^+^ and iROI-M^−^ in injured infants, with non-interferon-related differentially expressed genes in grey, and interferon-related differentially expressed genes in pink. **D** IRF8 + cells per mm^2^ in cROI (n = 7 infants), iROI-M^+^ (n = 5 infants) and iROI-M^−^ (n = 8 infants). Unpaired 2-tailed Student’s *t*-test, **P = 0.0046 for iROI-M^−^ versus cROI, θθ *P* = 0.0037 for iROI-M^−^ versus iROI-M^+^. **E** Representative images of IRF8 (green) and CD68 (red) double positive cells in injured regions (arrowheads), counterstained for Hoechst (blue). Scale bar, 20 µm. **F** Mean IRF8 + CD68 + cells/mm^2^ in cROI (n = 7 in fants), iROI-M^+^ (n = 5 infants) and iROI-M^−^ (n = 8 infants). Unpaired 2-tailed Student’s *t*-test, **P = 0.0046 for iROI-M^−^ versus cROI. **G** Mean IRF8 + CD68 + cells per mm^2^ in iROI-M^+^ versus iROI-M^−^. Paired 2-tailed Student’s *t*-test, *P = 0.0409, n = 4 infants. **H** Percentage of CD68 + cells which are IRF8 + in iROI-M^+^ versus iROI-M^−^. Paired 2-tailed Student’s *t*-test, *P = 0.0363, n = 4 infants. **I** Representative images of myelin (MBP; green) and microglia/macrophages (CD68; red) in infant with good myelination in iROI-M^−^ (infant 4) versus infant with poor myelination, counterstained for Hoechst (blue) in iROI-M^−^ (infant 3). Scale bar, 100 µm

The presence of iROI-M^+^ and iROI-M^−^ areas in the same samples gave us a unique opportunity to compare these to investigate mechanisms of myelination failure, while avoiding issues of inter-individual differences in genetic background and severity of injury (Fig. 2A). Of the 6 injured infant samples profiled by DSP, 4 had both iROI-M^+^ and iROI-M^−^ and were thus used for this comparative analysis. Differentially expressed genes (DEGs) between iROI-M^+^ and iROI-M^−^ were identified in all 4 cases (Injured1: 83 genes; Injured 2: 80 genes; Injured 3: 94 genes, Injured4: 39 genes) (Additional file 3: Sheet 2). Ingenuity Pathway Analysis of DEGs (Additional file 4: Sheet 3) identified significant regulation of pathways previously implicated in developmental myelination failure, such as Wnt signalling [10, 27, 39] (3/4 infants; ‘*Wnt/β-catenin signalling’, ‘Role of Wnt/GSKβ signalling in the pathogenesis of influenza’,* and* ‘Wnt/Ca2* + *pathway’*), the inflammasome pathway [13] (3/4 infants; ‘*IL1 signalling’*, and ‘*Inflammasome pathway’*), and ‘*IGF-1 signalling*’ [3] (2/4 infants). To identify a shared pathway amongst all 4 infants, we analyzed DEGs for common predicted upstream regulators and found that these related to Type II interferon (IFN-γ-associated) signalling, including IFN-γ itself (IFNG), and factors known to induce, or be a product of, this signalling (LPS and TNF, respectively; Fig. 2B; Additional file 4: Sheet 3). Consistent with this finding, between 40 and 49% of DEGs between iROI-M^−^ and iROI-M^+^ were interferon-related genes (Fig. 2C; Additional file 5: Sheet 4). Control infants generally had low interferon-related gene expression, although 3 interferon-related genes were upregulated in the 2 infants that died of Sudden Unexpected Death in Infancy (SUDI) versus other causes of death (Fig. S1E).

To validate the transcriptomic data, we assessed protein expression of Interferon Regulatory Factor 8 (IRF8), a master regulator of interferon responses in neurodegenerative and neuroinflammatory conditions, in all profiled infants and additional samples from an independent cohort (total n = 7 control infants, n = 8 injured infants; Additional file 1: Table S1). We found that IRF8 + cells were significantly increased in iROI-M^−^ versus iROI-M^+^ and cROI (Fig. 2D), demonstrating an enhanced interferon response in poorly myelinating regions. Only ~ 6% of IRF8 + cells were of the oligodendrocyte lineage (OLIG2 +), and this did not significantly change from iROI-M^−^ versus iROI-M^+^ or cROI (Additional file 1: Fig. S2A). We next assessed IRF8 expression in microglia/macrophages given their major roles in responding to inflammatory cues; whereas IRF8 is involved in microglia specification in early gestation, it is normally downregulated with microglial maturation by late gestation [16, 17], prior to the ages of cases assessed in our study. We found that CD68 + cells accounted for > 30% of IRF8 + cells in iROI-M^−^ (Additional file 1: Fig. S2B), and the density of IRF8 + CD68 + cells was increased in iROI-M^−^ relative to iROI-M^+^ and cROI (Fig. 2E, F). From all samples used for validation (DSP samples + additional cohort), 5 infants had both iROI-M^−^ and iROI-M^+^; of these, 4 infants had increased densities and percentages of IRF8 + CD68 + cells in iROI-M^−^ versus iROI-M^+^ (Fig. 2G, H). The one sample showing a decrease in IRF8 + CD68 + cells in iROI-M^−^ (Infant 4, 0d; Fig. 2G, H) was the only injured case in which there were myelin sheaths within injured regions despite short-term survival (Fig. 2I), with short sheaths reminiscent of regenerated myelin following damage (‘remyelination’). This was in stark contrast to the paucity of myelin sheaths in other injured sample iROI-M^−^, even with longer survival times (e.g. Infant 3, 21d; 2I). Therefore, enhanced Type II interferon signalling in microglia/macrophages is spatially associated with human developmental myelination failure.

### Increase in oligodendrocytes unable to appropriately form myelin in poorly developing white matter

**Fig. 3:**
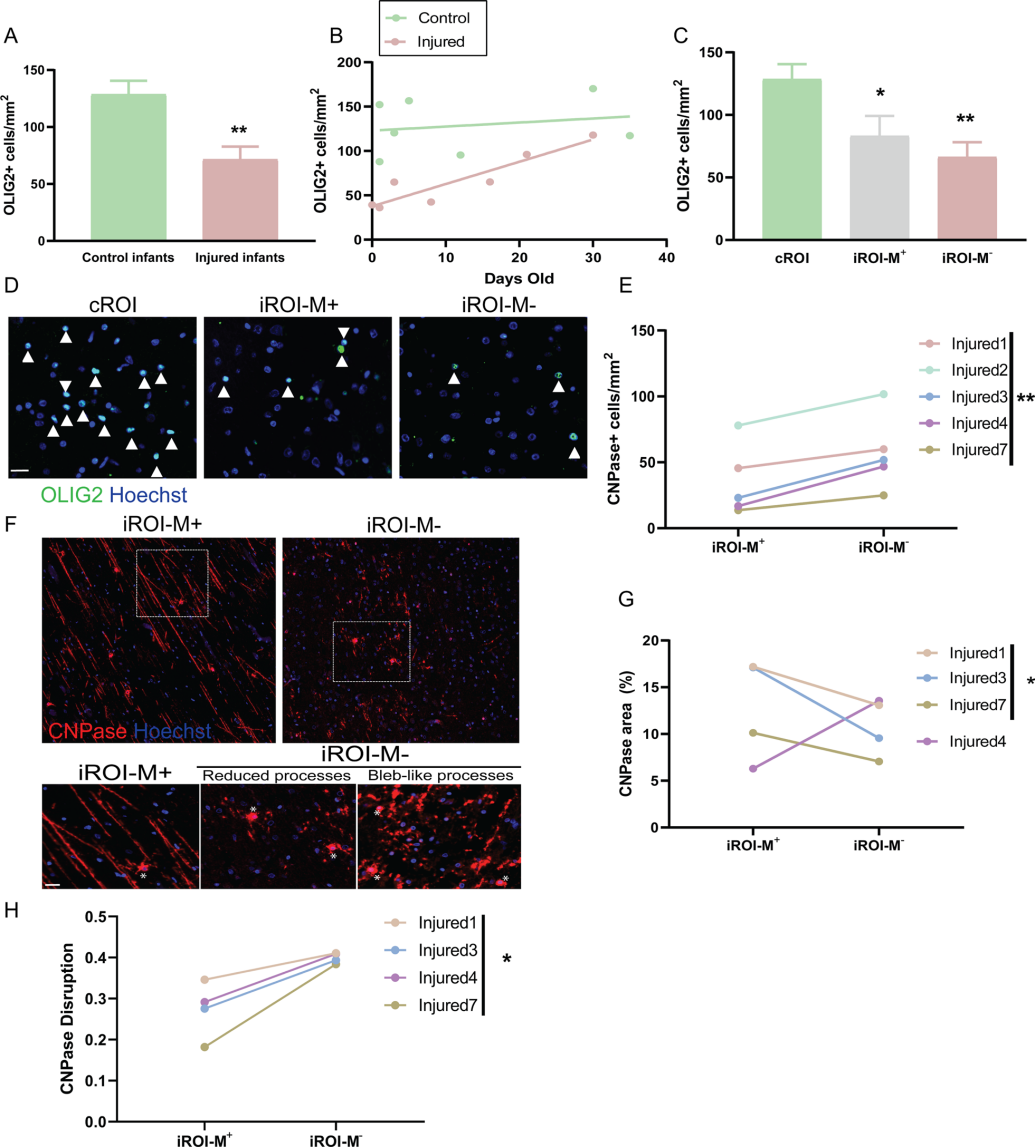
Oligodendrocyte lineage responses in poorly myelinating developing white matter. **A** Mean oligodendrocyte lineage cell (OLIG2 +) number per mm^2^ in control (n = 7 infants) and injured (n = 8 infants) cases. Unpaired 2-tailed Student’s *t*-test, **P = 0.0044. **B** OLIG2 + cells per mm^2^ in control (green) and injured (pink) infants versus age (days old), in infants born at term. N = 7 infants per group. **C** OLIG2 + cells per mm^2^ in cROI (n = 7 infants), iROI-M^+^ (n = 5 infants), versus iROI-M^−^ (n = 8 infants). Unpaired 2-tailed Student’s *t*-test, **P = 0.0028 for cROI versus iROI-M^−^, *P = 0.0423 for cROI versus iROI-M^+^. **D** Representative images of OLIG2 + cells (green) in cROI, iROI-M^+^, and iROI-M^−^, indicated by arrowheads. Nuclei counterstained with Hoechst (blue). Scale bar, 20 µm. **E** Mean CNPase + cells per mm^2^ in iROI-M^+^ versus iROI-M^−^. Paired 2-tailed Student’s *t*-test, **P = 0.0046. n = 5 infants. **F** Representative images of CNPase + cells (red) in iROI-M^+^ and in iROI-M^−^, showing reduced and bleb-like processes in the latter. Nuclei counterstained with Hoechst (blue). Cell bodies indicated by asterisks. Scale bar, 20 µm. **G** Percentage of CNPase + area in iROI-M^+^ versus iROI-M^−^ in term infants (excluding pre-term Infant 2). Paired 2-tailed Student’s *t*-test, *P = 0.0478, n = 3 infants. **H** CNPase Disruption as measured by sphericity in iROI-M^+^ versus iROI-M^−^ in term infants. Paired 2-tailed Student’s *t*-test, *P = 0.0215, n = 4 infants

We next asked how the dysregulated interferon signalling in microglia/macrophages impacted myelin-forming cells, the oligodendrocytes. We found that injured infants had an overall decrease in all oligodendrocyte lineage cells—encompassing progenitors through to mature oligodendrocytes—as identified by OLIG2 + immunofluorescence (Fig. 3A), and supported by a decrease in *SOX10* RNA expression (Additional file 1: Fig.S2C). Although OLIG2 + cell densities increased with age in injured infants, they were consistently reduced compared to age- and sex-matched controls (Fig. 3B). Both iROI-M^+^ and iROI-M^−^ showed decreased OLIG2 + cells compared to cROI (Fig. 3C, D), suggesting a global impact on the oligodendrocyte lineage, consistent with diffuse injury. However, there was no consistent difference in OLIG2 + densities between iROI-M^+^ and iROI-M^−^ (Additional file 1: Fig.S2D), therefore overall changes in total oligodendrocyte lineage cells could not explain the focal differences in myelination. To address this, we investigated cells of different maturation levels in the oligodendrocyte lineage. We found that oligodendrocyte progenitor cells (OPCs), identified by OLIG2 + cells negative for oligodendrocyte cell body marker CC1, were significantly reduced in iROI-M^−^ versus cROI (Additional file 1: Fig. S2E). We next investigated myelinating oligodendrocytes by assessing CNPase, which labels late oligodendrocyte lineage cell bodies and their extending myelin processes. We found that CNPase + cells were unexpectedly increased in iROI-M^−^ vesus iROI-M^+^(Fig. 3E). CNPase + cells formed myelin processes in all term infants (i.e. not in preterm Infant 2), yet their morphology differed. In iROI-M^−^, CNPase + cells extended fewer processes as measured by reduced CNPase area (Fig. 3F, G), with the exception of the aforementioned injured infant 4 that showed some myelination in iROI-M^−^ (Fig. 3G). In addition, processes had myelin disruption with an abnormal bleb-like morphology of CNPase staining (Fig. 3F, H), which was independent of process area or cell number (Additional file 1: Fig. S2F, G). Thus, surprisingly, our findings revealed that oligodendrocyte maturation is not impaired in poorly myelinating regions of infant white matter; rather, oligodendrocytes with myelinating potential are relatively increased in number yet unable to appropriately form myelin.

### Interferon-responsive microglia functionally impede myelin maturation by mature oligodendrocytes

**Fig. 4:**
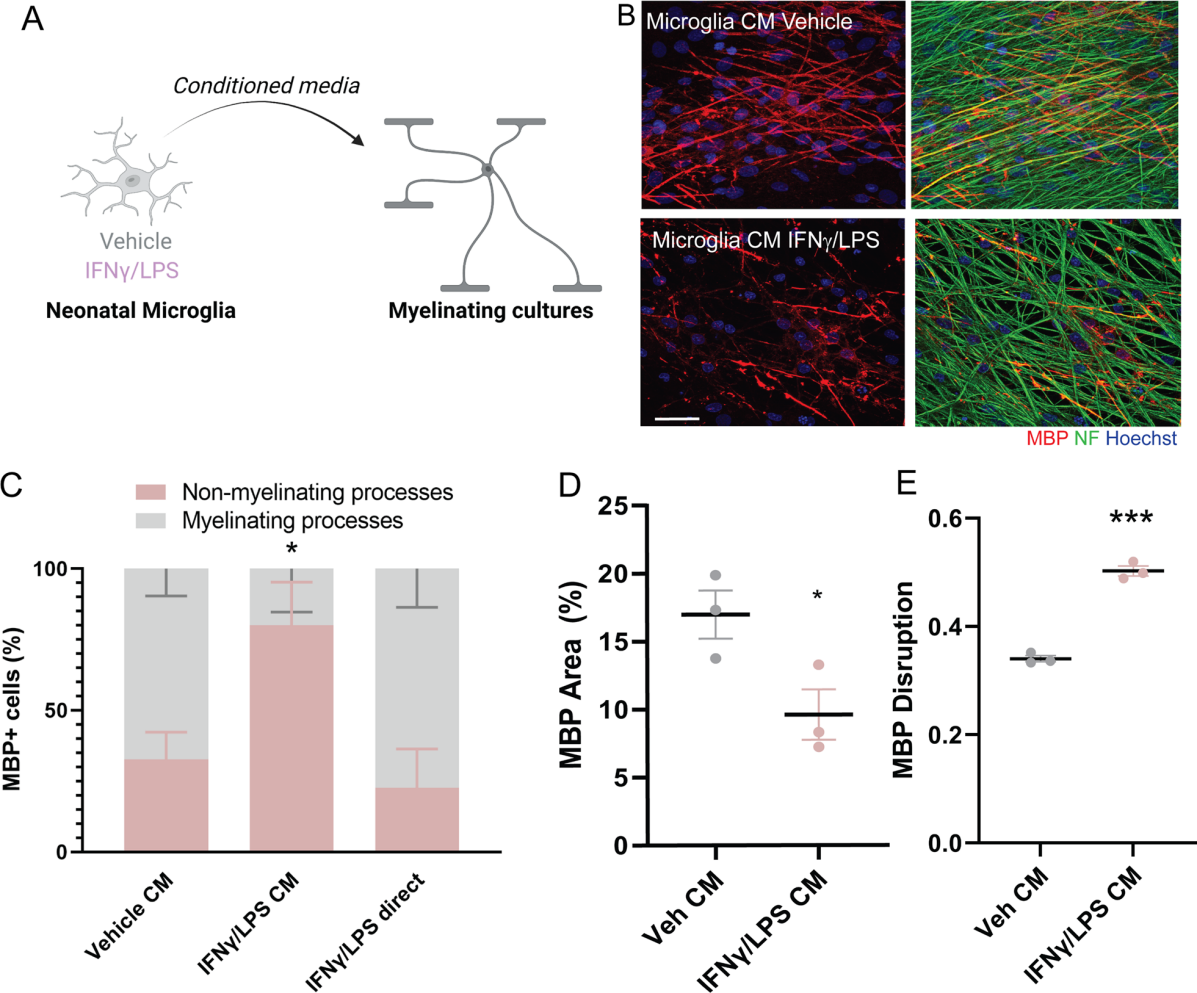
Interferon-responsive microglia functionally impede myelination by mature oligodendrocytes. **A** Diagram of treatment of neonatal microglia cultures with IFNγ and LPS, or vehicle control, and application of conditioned media to myelinating cultures of oligodendrocytes and neurons. **B** Representative images of cultures exposed to conditioned media (CM) from vehicle (PBS)-treated microglia or IFNγ and LPS-treated microglia, with myelin indicated by MBP (red), axons indicated by NF (green), and nuclei counterstained with Hoechst (blue). Scale bar, 25 µm. **C** Percentage of oligodendrocytes (MBP +) which have myelinating processes (grey) versus non-myelinating processes (pink) following treatment with CM from vehicle-treated or IFNγ/LPS-treated microglia, or with IFNγ/LPS directly applied to myelinating cultures. Unpaired 2-tailed Student’s *t*-test, *P = 0.0428 for non-myelinating processes, and *P = 0.0428 for myelinating processes, for IFNγ/LPS CM versus Veh CM. N = 3 biologically independent repeats. **D** Percentage of MBP area in myelinating cultures exposed to vehicle (Veh) or IFNγ/LPS-treated microglia CM. Unpaired 2-tailed Student’s *t*-test, *P = 0.0455. N = 3 biologically independent repeats. **E** MBP Disruption as measured by sphericity in myelinating cultures exposed to Veh or or IFNγ/LPS treated microglia CM. Unpaired 2-tailed Student’s *t*-test, ***P = 0.0001. N = 3 biologically independent repeats

We then tested whether there was a functional link between our two findings of increased Type II interferon-signalling in microglia/macrophages and the inability of mature oligodendrocytes to form proper myelin. As in vivo overexpression of IFNγ in the CNS during early development (when myelination normally takes place) causes confounding cerebellar tumours and maldevelopment [7, 20], we instead turned to a simplified in vitro system. We treated isolated cultures of primary neonatal rat microglia with IFNγ and a known potentiator of interferon signaling, lipopolysaccharide (LPS), and added their conditioned media (CM) to myelinating co-cultures of oligodendrocytes and neurons (Fig. 4A). Following exposure to CM from vehicle-treated microglia, the majority of mature oligodendrocytes (MBP + cell bodies) formed myelinating processes aligned with axons (NF + ; Fig. 4B, C). Conversely, exposure to IFNγ/LPS-treated microglia CM increased the proportion of oligodendrocytes without myelinating processes (Fig. 4B, C) and decreased myelin sheath formation (reduced MBP area; Fig. 4D). There was no deleterious impact of IFNγ/LPS provided directly to myelinating cultures (Fig. 4C), and our previous work excluded direct effects of these factors on oligodendrocyte lineage cell proliferation, survival, and differentiation [25]. Of note, we did not observe a change in total oligodendrocyte lineage number (7.2 ± 5 cells/field in vehicle CM versus 10.2 ± 6 cells/field in IFNγ/LPS CM), suggesting that the decrease in the total oligodendrocyte lineage observed in myelinating and non-myelinating areas of injured human brain may be regulated via a distinct mechanism. The limited myelin sheaths formed in the IFNγ/LPS-treated microglia CM condition had myelin disruption with an abnormal bleb-like morphology of MBP staining (Fig. 4E), which was independent of MBP area (Additional file 1: Fig. S2H) and was similar to the abnormal myelin sheaths seen in poorly myelinating human white matter. Together, these findings indicate that enhanced Type II interferon signalling in microglia is sufficient to impede appropriate myelin maturation by mature oligodendrocytes.

To address whether interferon-responsive microglia themselves release IFNγ to influence oligodendrocytes, we performed proteomic analysis of nascent proteins in the microglia conditioned media, by exposure of cells to the labelled amino acid analogue AHA at the time of treatment followed by liquid chromatography/tandem mass spectrometry of labelled peptides. Although pro-inflammatory cytokines TNFα and IL1β were enriched in IFNγ/LPS-treated microglia CM, as we have previously found [25], IFNγ was not detected (Additional file 1: Fig. S2I; Additional file 6: Sheet 5). Thus, interferon-responsive microglia in turn influence myelinating oligodendrocytes via an IFNγ-independent mechanism.

### Osteopontin (SPP1) is a biomarker of poor myelination in human developing white matter

**Fig. 5:**
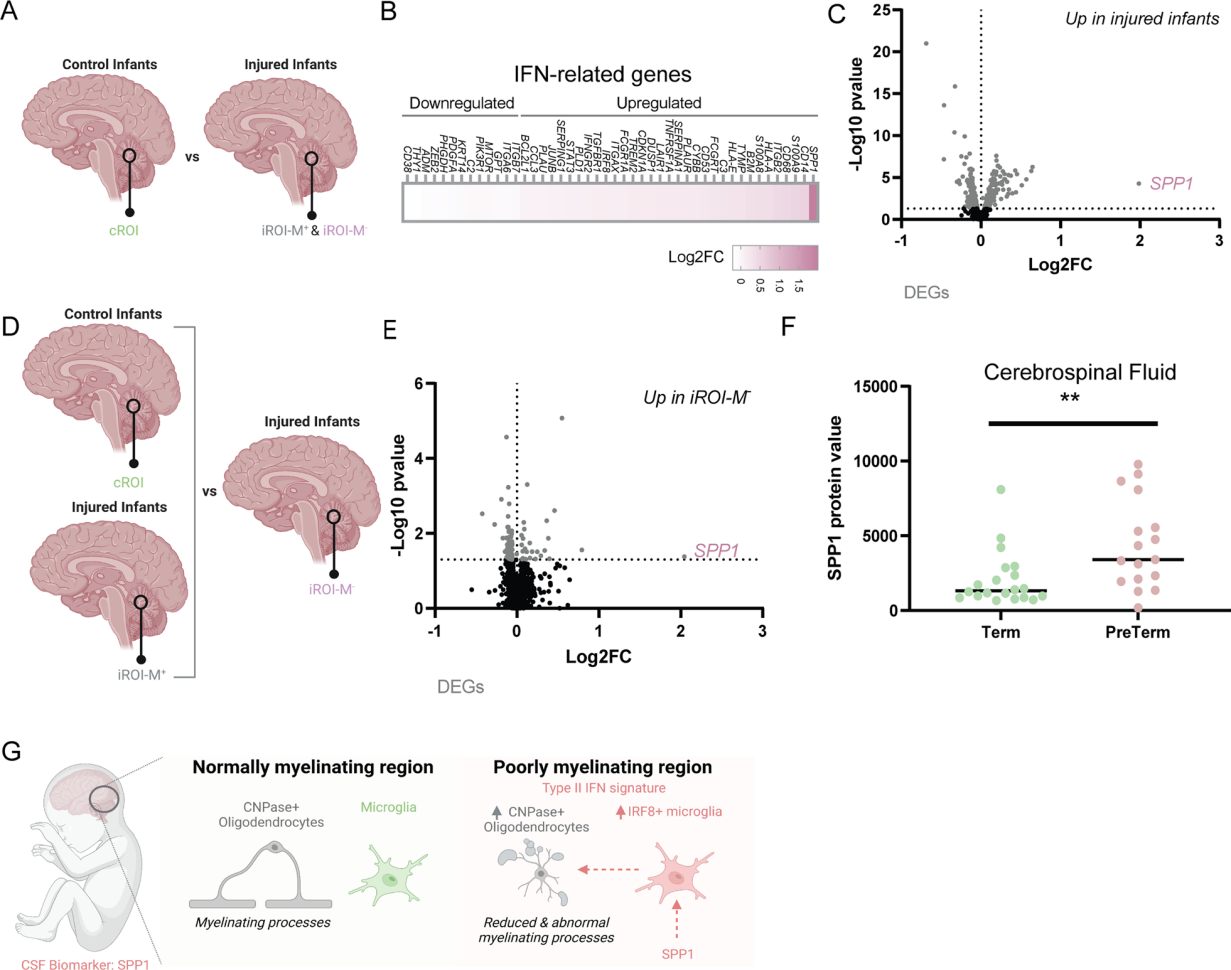
Osteopontin as a biomarker for poor myelination in developing white matter. **A** Diagram of comparison between control infant iROI (cROI) and injured infant ROI (iROI-M^+^ and iROI-M^−^). **B** Heatmap of IFN-associated DEGs in injured versus control infants, indicated as Log2FC. **C** Volcano plot of DEGs in injured versus control infants indicated in grey. Osteopontin (*SPP1*) is indicated in pink (*P* = 0.0005). N = 6 control infants and 6 injured infants. **D** Diagram of comparison between normally myelinating areas (control infant cROI and injured infant iROI-M^+^) versus poorly myelinating areas (injured infant iROI-M^−^). **E** Volcano plot of differentially expressed genes in iROI-M^−^ versus cROI/iROI-M^+^, indicated in grey. Osteopontin (*SPP1*) is indicated in pink (*P* = 0.04213). N = 6 control infants and 6 injured infants. **F** Levels of SPP1 protein in cerebrospinal fluid of term controls and infants at high risk of brain white matter damage (preterm). Mann–Whitney test, **P = 0.0031, n = 20 term infants and 17 preterm infants. **G** Graphical abstract: Compared to normally myelinating regions of infant brain, poorly myelinating regions have a Type II interferon signature associated with increased interferon-responsive microglia (IRF8 +), increased CNPase + oligodendrocytes which produce less myelin and abnormal myelin, and increased interferon-inducer SPP1—also detectable in cerebrospinal fluid of infants at high risk of brain injury

We next asked whether we could detect a related biomarker for poor myelination, by comparing transcriptomes of injured infants to those of control infants (Fig. 5A). We identified 467 DEGs, which included upregulation of 3 genes in injured infants previously associated with developmental white matter injury in rodents: *LGALS3*, *CD44*, and *TGFB1* [5, 8, 28] (Additional file 7: Sheet 6). Unbiased pathway analysis indicated differential regulation of interferon signalling (89/467 genes in ‘*Interferon gamma signalling*’, *P* = 1.11 × 10^–16^; 102/467 genes in ‘*Interferon signalling*’, *P* = 8.42 × 10^–14^). Ten percent of the DEGs overlapped with the interferon-related DEGs from the comparison between iROI-M^+^ and iROI-M^−^, including *IRF8* and the gene encoding the IFN-γ receptor *IFNGR2* (Fig. 5B; Additional file 7: Sheet 6). Notably, the most upregulated of all DEGs and IFN-related genes in injured infants was *SPP1* (Osteopontin) (Log2 fold change = 1.98; Fig. 4B, C), of interest as it is known to induce Type II interferon signalling and is expressed by immune and neural cells following experimental insult [19, 35]. *SPP1* was also the most upregulated gene when comparing iROI-M^−^ to all other ROIs (cROI and iROI-M^+^) suggesting that it associates with poorly myelinating areas (Fig. 5D, E; Additional file 8: Sheet 7). As SPP1 is a secreted protein, to validate these findings we assessed levels in the cerebrospinal fluid (CSF). Due to the lack of an existing cohort with matched post-mortem brain tissue and CSF, we mined a pre-existing protein database of CSF collected from surviving infants [4], comparing healthy term infants to preterm infants, the latter a demographic at high risk of developmental myelination failure (see Additional file 1: Table S2). We observed a significant increase in SPP1 CSF protein levels in preterms compared to healthy term controls (Fig. 5F). As CSF collection occurred at time of clinical need, infants were not age-matched between the groups; however, *SPP1* RNA was significantly increased in both preterm and term injured infants, suggesting that prematurity alone is not sufficient to elevate levels. *SPP1* gene expression showed sex differences, being the most upregulated gene in females versus males in our samples regardless of injury or region (Additional file 1: Fig. S3A–C), however this was not observed when analysing CSF protein levels (Additional file 1: Fig. S3D). Of note, SPP1 was not enriched in conditioned media from interferon-responsive microglia versus vehicle-treated microglia (Additional file 1: Fig. S2I), suggesting an alternate source of SPP1. In summary, elevated SPP1 represents a potential biomarker for poor myelination in human infants.

## Discussion

In this study, we identified the molecular signature of myelination failure in human developing CNS, using digital spatial transcriptomics of a rare brain bank to uncover a localized dysregulated microglial response associated with poor myelination (Fig. 5G). We found that an enhanced Type II interferon (IFNγ) response by microglia is associated with spatial specificity in reduced and abnormal myelin processes formed by oligodendrocytes. This has three important implications.

First, our work suggests the importance of Type-II interferon signaling in human developmental myelination failure. Of interest, IFN-γ is known to influence the expression or activity of other factors/pathways implicated in this process, such as Wnt, the NLRP3 inflammasome, and PSD-95 [10, 13, 18, 27, 39], pointing to a potential new role as a master regulator. Although altered pro-inflammatory cytokine levels have been documented in injured infants [4, 11, 30], the weight of contribution of individual factors to cellular responses and pathology are unknown; indeed, no single inflammatory pathway has been predominantly implicated. Our comprehensive and unbiased analysis indicates that the majority of transcriptomic changes associated with myelination failure are associated with IFNγ signaling, and we demonstrate that microglia/macrophages are major responders to this signal in this context. We further discovered that interferon-responsive microglia are sufficient to dysregulate myelin maturation in vitro. Interestingly, our data associating Type II-interferon (IFNγ) responsive microglia with disrupted developmental myelination contrasts with the regenerative role of Type I-interferon (IFN-α, β) signalling in microglia during myelin regeneration in adulthood [21], likely reflecting differential downstream signaling, stage of microglia maturation, age and microenvironment. The cellular source of IFNγ in the context of human developmental myelination failure is unknown. T lymphocytes are potential candidates given their production of IFNγ in association with interferon-responsive microglia in ageing white matter [15]. Although our transcriptomic analysis suggests a small but significant upregulation of T cell-associated genes (*CD4, CD8*) in injured versus control infants (Additional file 7: Sheet 6), the lack of differential expression between hypomyelinating and normally myelinating regions in injured infants (Additional file 3: Sheet 2) makes it unclear as to whether there is significant lymphocyte infiltration.

Second, we unexpectedly found that late oligodendrocyte lineage cells are not only present, but are increased in poorly myelinating areas relative to adjacent normally myelinating areas. This finding suggests an uncoupling of oligodendrocyte maturation from myelin pathology, a phenomenon not observed in in vivo experimental models of developmental white matter injury where impaired oligodendrogenesis underpins hypomyelination. This critical difference in pathological responses to white matter injury in human versus in vivo rodent CNS highlights the importance of investigating human tissue. Although we cannot discount impaired oligodendrocyte maturation in otherwise normal-appearing human infant white matter—which may account for the global decrease in oligodendrocyte lineage cells compared to controls—this is not associated with impaired myelination in the human brain. However, the consequence of this global oligodendrocyte lineage cell depletion on long-term myelin health is unknown. Interestingly, we discovered that oligodendrocytes in injured regions are unable to form myelin appropriately, highlighting the potential interaction between these mature cells and microglia. This may suggest that existing mature oligodendrocytes may be novel therapeutic targets to be manipulated to enhance myelination in development. Though mature oligodendrocytes have only recently been shown to occasionally form new myelin following CNS damage in adulthood [2], whether this can occur following developmental injury is unknown, and the poor quantity and targeting of this myelin [26] highlights the need to better understand mechanisms that would encourage healthy axonal ensheathment after injury. We also identified a significant reduction in OPCs in hypomyelinating regions compared to control infant white matter, representing either a deficit in oligodendrocyte lineage specification, cell death, or increased maturation into non-myelinating oligodendrocytes. Interestingly, our previous work showed that interferon-responsive microglia conditioned media increases, rather than decreases, OPC numbers [25], suggesting a distinct pathological mechanism regulating OPCs in the context of human myelination failure.

Third, we uncovered the upregulation of Osteopontin (SPP1) in injured human developing white matter areas, complementing its upregulation in adult neurodegenerative disorders [22, 32, 37, 38]. Its known roles in regulating Type II interferon signaling suggest it could be a useful biomarker for developmental myelination failure. The function of Osteopontin in human myelination failure is unknown and requires further investigation, though in rodent models Osteopontin has been associated with both a pro-myelinating/ neuroprotective function and a worsening of white matter injury, reflecting differing forms of the protein, injury context, and age [1, 6, 19, 35, 41]. Species-specific differences may need to be considered, as Osteopontin is not detectable outside of the brain of rodents following developmental CNS injury [19], in contrast to our findings in the human infant CSF. Our study highlights the need for long-term follow-up of cohorts linking biomarker analysis with brain imaging and development.

## Conclusions

By conducting a comprehensive transcriptomic analysis of human developing white matter together with protein validation and functional experimentation, our study suggests dysregulated microglia-oligodendrocyte interaction via Type II interferon signaling to be a critical regulator of myelination failure in human CNS.

## Materials and methods

### Human infant samples

Formalin-fixed paraffin-embedded (FFPE) human infant sections. (10 µm) of cerebellum were obtained through Brain UK (The University of Southampton), under licenced approval from South Central- Hampshire B Research Ethics Committee (Ref:14/SC/0098, 19/SC/0217, 13/006). Tissue was anonymised by Brain UK prior to use. Sex, age, cause of death, and neuropathological assessment can be found in Additional file 1: Table S1. Only cases with a pathological diagnosis of white matter damage from periventricular leukomalacia or hypoxia/hypoxic ischemic encephalopathy were included in this study. Human infant CSF was collected for a prior study and details of cases are as previously described [4]; clinical information can be found in Additional file 1: Table S2.

### Digital spatial profiling

Tissue transcriptomes were evaluated using the GeoMx Cancer Transcriptome Atlas with 1825 RNA targets. Spatial transcriptomics analysis included 12 ROIs per tissue (650 µm × 650 µm). Slides were incubated at 67 °C for 1 h and loaded into Leica Biosystems BOND RX FFPE for deparaffinization/ rehydration. Slides were then treated with 0.1 µg/mL proteinase K solution to expose RNA targets then fixed with 10% Neutral Buffered Formalin. Slides were incubated overnight with RNA probe mix (> 8000 in situ hybridization probes with UV photocleavable oligonucleotide barcodes), then washed and stained with mouse anti-MBP (Novus; NBP2-22121AF594, 2H9), goat anti-CD68 (Santa-Cruz; sc-20060AF647, KP1), and Syto83 (Thermo Scientific). Slides were loaded onto the GeoMx DSP instrument and scanned to obtain whole-tissue images, and serial UV illumination of each compartment was used to sequentially collect the probe barcodes from the different ROIs identified based on fluorescent information on myelination and inflammation. Once all ROIs completed 18 cycles of PCR (MAN-10133-03) and were purified into a single pooled library, this was then sequenced by the Illumina NovaSeq 6000 system. Samples were indexed to generated over 1.8 billion reads in total, demultiplexed and converted to digital count conversion files, and converted to an expression count matrix. The 75th percentile of the gene counts for a given gene was calculated for each ROI, and normalized to the geometric mean of the 75th percentile across all ROIs to give the upper quartile (Q3) normalization factors for each ROI. Two of the 144 ROIs were removed from analysis due to under-sequencing (one from a control infant, one from an injured infant).

### Analysis of DSP data

Differentially expressed gene lists and Principal Component scores were generated using the GeoMx DSP Control Centre software v2.5.0.145 (Nanostring Technologies, Inc.), using *t*-test or linear mixed model with a threshold of *P* < 0.05 to generate a list of differentially expressed genes. These genes were inputted into pathway analysis software (Ingenuity Pathway Analysis (IPA) and Reactome) to generate hypotheses on significantly regulated pathways and predicted upstream regulators.

### Immunohistochemistry of human tissue sections

Sections were preheated at 60 °C for 10 min before deparaffinisation at room temperature in Histoclear and rehydration through an ethanol gradient. Following a wash in Tris-buffered Saline (TBS) with 0.001% Triton X-100 (Sigma), sections were microwaved in antigen unmasking solution (pH6 citrate buffer, Vector Laboratories) for 10 min, then heated at 60 °C for 30 min. After cooling, sections were washed once with TBS and 0.001% Triton X-100, and endogenous phosphatase and peroxidase activity blocked with Bloxall (Vector) for 10 min. Blocking was performed for 1 h with 10% Heat Inactivated Horse Serum (Gibco) and 0.5% Triton X-100 in TBS. Primary antibody diluted in blocking solution was applied overnight in a humid chamber at 4 °C. Antibodies used include rat anti-MBP (1:250, Bio-Rad), mouse anti-CD68 (1:100, DAKO), rabbit anti-IRF8 (1:100, Thermo Fisher Scientific), mouse anti-OLIG2 (1:100, EMD Millipore), rabbit anti-OLIG2 (1:100, EMD Millipore), mouse anti-CNPase (1:250, Atlas Antibodies), and mouse anti-APC (CC1; 1:100, Abcam). Following 3 washes in TBS with 0.001% Triton X-100, peroxidase-conjugated secondary antibody (Vector) was applied for 1 h at room temperature in a humid chamber. Following further washes, sections were developed by using Opal 520 (Akoya) at 1:100 in Plus Amplification Diluent (Akoya) for 10 min in a humid chamber, washed, and residual peroxidase activity was quenched by applying Bloxall (Vector) for 10 min. For co-staining, another primary antibody was then applied and developed using peroxidase-conjugated secondary antibody and Opal 570 (Akoya) at 1:100 in Plus Amplification Diluent as described above, or by using an Alexa-555 conjugated secondary antibody (Invitrogen). Following washes in water, the sections were counterstained with Hoechst (1:10,000) and mounted with Fluoromount-G (Invitrogen).

### Tissue imaging and analysis

Entire tissue sections were imaged using a Zeiss AxioScan SlideScanner. Regions of interest (ROI) were defined for analysis using Zeiss Zen 3.3 (Blue edition) software and were matched to those used for spatial profiling. Comparisons were made between healthy control cases (cROI) and injured infants (iROI), or between normally myelinating regions versus regions with reduced myelination in injured infants (iROI-M^+^ and iROI-M^−^, respectively). At least 3 representative fields of 806 µm × 662 µm were assessed per type of ROI per sample. Density of immunopositive cells per mm^2^ was counted in a blinded manner. Area of CNPase or MBP was obtained using ImageJ to measure percentage of area covered by positive pixels. Myelin disruption was quantified by CNPase or MBP sphericity, analysed using the surface measuring function in Imaris 9.8 software.

### Microglia isolation and treatment

All animal work was carried out under a project licence from the United Kingdom Home Office in accordance with ARRIVE2 guidelines. Cortices from Sprague Dawley rat postnatal day 0–2 pups (both male and female) were used to generate mixed glial cultures. At 10 days of culture, microglia were isolated by collecting the floating fraction following 1 h on a rotary shaker at 37 °C at 250 rpm, and plated in Dulbecco’s modified essential media (DMEM) containing 4.5 g/l glucose, l-glutamine, pyruvate, 10% fetal calf serum, and 1% penicillin/streptomycin (GIBCO) on poly-d-lysine (PDL)-coated glass coverslips at 1 × 10^6^ cells per well in a 24-well plate. Microglia were either treated overnight with IFNγ (20 ng/mL) and LPS (0127:B8, 100 ng/mL) (all from Sigma-Aldrich; < 1 EU/μg) or PBS vehicle control. Conditioned media was collected and stored at − 20 °C for later application to myelinating cultures.

### Proteomics of nascent proteins in microglia conditioned media

Primary microglia were isolated and plated as above, except at double the density in DMEM devoid of glutamine, methionine, and cysteine supplemented with 10% fetal calf serum and 1% penicillin/streptomycin. Cells were co-treated with PBS or IFNγ and LPS (as above) together with AHA (100 µM; Life Technologies ‘Click-It AHA’). Conditioned media was collected 1 day later, supplemented with protease inhibitor and stored at − 80 °C. The experiment was repeated with 3 independent litters. Newly-synthesized AHA-labeled proteins were enriched in conditioned media using the Click-iT^®^ Protein Enrichment Kit (Invitrogen C10416) according to manufacturer’s instruction with optimized media to resin and to catalyst ratio. Before setting up the reaction, 0.9 mL of conditioned media was first concentrated to 0.5 mL in urea lysis buffer using MWCO filter device (Amicon Ultra^®^ Centrifugal Filters, 3-kDa cutoff, 0.5 mL, Millipore, Billerica, MA); 2 mL of alkyne agarose resin (50% slurry) was washed following instruction and left in approximately 1 mL in the tube, and 1 mL of 2X catalyst solution was freshly prepared following instruction. After an 18 h reaction (head-over-end rotation) at room temperature, dithiothreitol reduction and Iodoacetamide alkylation of resin-bound protein, stringent washing of the resin, and digest of the Resin-Bound Proteins were all performed according to the manufacturer’s instruction. Desalt of the digest was performed using C18 tips (Agilent Bond Elut Omix 18, Cat No. A57003100) on DigestPro (Intavis, Koln, Germany). The eluate containing desalted peptides was dried and stored in − 20 °C until analysis. Dried peptide extracts were reconstituted in 5% acetonitrile, 0.2% formic acid and subsequently analyzed on nanoLC-MS/MS platform composed of Eksigent nLC 2D plus (AB Sciex) and Q Exactive mass spectrometer (Thermo Fisher). Peptides were separated on nano C18 column (ProteoPep II, 15 cm × 75 µm, 5 µm, 300 Å, New Objective, Woburn, MA) at flowrate of 300 nL/min (solvent A: 0.1% formic acid; solvent B: acetonitrile, 0.1% formic acid) with a 94 min gradient (from 5 to 15% B at 3 min, to 21%B at 45 min, to 35% B at 85 min, to 50% B at 89 min, and finally to 75% B at 94), and analyzed by mass spectrometer at data dependent mode (400–1800 m/z range, isolation window 3 m/z, dynamic exclusion 20 s; resolution, AGC target and maximum injection time are 35,000, 1e6, and 80 ms for MS1, 17,500, 1e5, and 120 ms for MS2). Mass spec data were searched using Mascot (version 2.4) against swissprot rat and bovine databases (August 2013 release), with 20 ppm (MS1) and 50 mmu (MS2) mass accuracy, cysteine carbamidomethylation as a fixed modification, and methionine oxidation and replacement of methionine by AHA as variable modifications. Secreted rat proteins were annotated based on the compendium of human secreted proteins [9].

### Myelinating cultures

Dorsal root ganglia were plucked from E15 Sprague–Dawley rat spinal cords, chemically dissociated with papain (30 units/mL, final concentration 4%) and DNase I (40 μg/mL) for 1 h at 37 °C and mechanically dissociated by gentle trituration. Neurons were plated as a drop at 1.5 × 10^4^ cells per well on PDL- and matrigel-coated glass coverslips in a 12-well plate, in DMEM supplemented with 10% FCS, 1% penicillin–streptomycin and nerve growth factor (NGF; 100 ng/mL, AbD Serotec, Raleigh, NC). The following day, wells were flooded with media as above and underwent 3 rounds of treatment with a mitotic inhibitor FdU (10 μM; Sigma-Aldrich). Oligodendrocyte progenitor cells (OPCs) were isolated from mixed glial cultures by collecting the floating fraction following depletion of microglia as above then a 16–20 h shake-off on a rotary shaker at 37 °C at 250 rpm. Subsequently, astrocytes were depleted from the floating fraction by differential adhesion. OPCs were plated onto dorsal root ganglia at 21 days of neuronal culture at 7.5 × 10^3^ cells per well in Basal Medium Eagle (BME) supplemented with 0.5% FCS (GIBCO), insulin–transferrin–sodium selenite mix, 0.45% glucose, 1% penicillin–streptomycin and 1% Glutamax (all from Sigma-Aldrich). At 7 days of co-culture and at the initiation of myelination, co-cultures were either treated with conditioned media from vehicle-treated or IFNγ/ LPS-treated microglia in a 1:1 ratio with culture media for 7 additional days. Data represents 3 biologically-independent experiments treated with conditioned media from at least 3 microglia preparations. Up to four 40X objective images were taken per condition per experiment for quantification.

### Immunocytochemistry of myelinating cultures

Following fixation with 4% paraformaldehyde (Sigma-Aldrich) for 10–15 min, coverslips were stored at 4 °C until immunostaining. Coverslips were blocked for 30 min in 5% normal horse serum (GIBCO) and 0.3% Triton-X-100 at room temperature, and primary antibodies were diluted in block and applied overnight at 4 °C in a humid chamber. Primary antibodies included rat anti-MBP (AbD Serotec, MCA409S, 1:250), and chicken anti-neurofilament-H (Encor Biotechnology, Inc., CPCA-NF-H, 1:10,000). Fluorescently-conjugated secondary antibodies were applied for 1 h at room temperature in a humid chamber (1:1000, Invitrogen). Following counterstaining with Hoechst (5 µg/mL), coverslips were mounted to slides using Fluoromount-G (Invitrogen).

### Statistical analysis

All manual cell counts were performed in a blinded manner. Data are represented as mean ± standard error of the mean (SEM). Comparisons of immunostains were made using 2-tailed paired or unpaired Student’s *t*-test. Comparison of CSF protein data was made using the Mann–Whitney test. *P* values of < 0.05 were considered statistically significant. Data handling and statistical processing was performed using Microsoft Excel and GraphPad Prism Software versions 8 and 9.

## Supplementary Information

**Additional file 1: Table S1.** Neuropathological and Clinical information of human infant brain samples used for digital spatial profiling and immunofluorescent assessment. **Table S2.** Clinical information of human infant cerebrospinal fluid samples used for SPP1 assessment. **Fig. S1.** Digital Spatial Transcriptomic profiling of human infant brain. **A** Overview images of ROI selection in injured infant cerebellum, stained for myelin (MBP; green), microglia/macrophages (CD68; red), and counterstained with Hoechst. **B** PCA plot of ROIs from female (orange) versus male (blue) human infant cerebellar white matter. **C** PCA plot of ROIs from human infant cerebellar white matter from cases with a neuropathological diagnosis of periventricular leukomalacia (PVL; purple) or hypoxic ischemic encephalopathy (HIE) or hypoxia (blue). Control infants are indicated in grey. **D** PCA plot of ROIs from human infant cerebellar white matter with age indicated. Individual infants indicated in different colours (injured are pink, control are green). **E** Volcano plot of control infants comparing those who died from Sudden Unexpected Death in Infancy (SUDI) versus other causes of death. Differentially expressed genes shown in grey, and interferon-related genes indicated in pink. **Fig. S2.** Oligodendrocyte lineage cell responses in human infant brain injury. **A** Mean proportion of IRF8+ cells that are OLIG2+ ± s.e.m. in cROI (n = 6 infants), iROI-M^+^ (n = 4 infants) and iROI-M^−^ (n = 6 infants). **B** Mean proportion of IRF8+ cells that are CD68+ ± s.e.m. in cROI (n+6 infants), iROI-M^+^ (n = 4 infants) and iROI-M^−^ (n = 6 infants).**P = 0.0039, Unpaired 2-tailed Student’s *t*-test. **C** Mean *SOX10* normalized RNA counts ± s.e.m. for control and injured infants. Unpaired 2-tailed Student’s *t*-test, **P = 0.0080, n = 6 infants per group. **D** Mean OLIG2+ cells per mm2 ± s.e.m. in iROI-M^+^ and iROI-M^−^. n = 5 infants. Unpaired 2-tailed Student’s *t*-test, *P *> 0.05. **E** Mean OLIG2+ CC1- cells per mm2 ± s.e.m. in cROI (n = 6 infants), iROI-M^+^ (n = 4 infants) and iROI-M^−^ (n = 6 infants). *P = 0.0108, Mann-Whitney test. **F** Sphericity (based on CNPase) versus percent CNPase area per respective ROI, in iROI-M^+^ (grey) and iROI-M^−^ (pink). **G** Sphericity (based on CNPase) versus number of CNPase+ cells per respective ROI, in iROI-M^+^ (grey) and iROI-M^−^ (pink). **H** Sphericity (based on MBP) versus MBP area in myelinating cultures exposed to conditioned media (CM) from microglia treated with PBS vehicle (grey) or IFNγ and LPS (pink). **I** Average fraction of total spectral counts (SC) in conditioned media (CM) from microglia treated with IFNγ and LPS, where proteins enriched in this condition are indicated in pink (e.g. TNF, IL1B) whereas proteins enriched in vehicle (Veh) control are indicated in grey (E.g. SPP1). **Fig. S3.** Analysis of sex and Osteopontin regulation in human infant brain injury. **A** Volcano plot of control infants, comparing female versus male gene expression. Differentially significantly regulated genes shown in grey. SPP1 indicated in pink. **B** Volcano plot of iROI-M^+^, comparing female versus male gene expression. Differentially significantly regulated genes shown in grey. SPP1 indicated in pink. **C** Volcano plot of iROI-M^−^, comparing female versus male gene expression. Differentially significantly regulated genes shown in grey. SPP1 indicated in pink. **D** SPP1 protein levels in CSF of term versus preterm infants, with females indicated in pink and males indicated in grey. Mann-Whitney test, **P = 0.0031, n = 20 term infants and 17 pre-term infants.

**Additional file 2: Sheet 1.** Digital spatial transcriptomic profiling of human infant brain injury. Normalized RNA counts for 1825 targets, for 12 ROI per sample. N = 6 control infants, 6 injured infants. ROI: region of interest. cROI: ROI from control infants. iROI-M^+^: ROI from normally myelinating regions of injured infants. iROI-M^−^: ROI from hypomyelinating regions of injured infants.

**Additional file 3: Sheet 2.** Differentially expressed genes in respective iROI-M^−^ versus iROI-M^+^. Differentially expressed genes (DEG) in respective iROI-M^−^ versus iROI-M^+^ in 4 injured infants. FC: fold change.

**Additional file 4: Sheet 3.** Pathway analysis of DEGs from respective iROI-M^−^ versus iROI-M^+^. Ingenuity Pathway Analysis (IPA) canonical pathways and predicted upstream regulators that are significantly regulated in comparison of respective iROI-M^−^ to iROI-M^+^ in 4 injured infants.

**Additional file 5: Sheet 4.** Interferon-related DEGs from respective iROI-M^−^ versus iROI-M^+^. Lists of interferon-related DEGs in iROI-M^−^ versus iROI-M^+^ in 4 injured infants.

**Additional file 6: Sheet 5.** Proteomic analysis of microglia conditioned media. Average spectral counts and spectral count fraction following treatment with IFNγ/LPS or vehicle control. n = 3 biological replicates per treatment condition.

**Additional file 7: Sheet 6.** Differentially expressed genes from comparison of injured infants to control infants. Differentially expressed genes (DEGs) and interferon-related DEGs in injured versus control infants. N = 6 control infants, 6 injured infants. FC: fold change.

**Additional file 8: Supplemental Sheet 7.** Differentially expressed genes from comparison of iROI-M^−^ to other ROI. Differentially expressed genes (DEGs) in all iROI-M^−^ versus all other ROI (cROI and iROI-M^+^ combined). N = 6 infants with iROI-M^−^, 6 infants with cROI, and 4 infants with iROI-M^+^.

## Data Availability

All DSP data and related analyses are included in this published article and its supplementary information files. All other data is available from the corresponding author upon request.

## Abbreviations

CM: Conditioned media
CNPase: 2ʹ,3ʹ-Cyclic nucleotide 3ʹ-phosphodiesterase
CNS: Central nervous system
CSF: Cerebrospinal fluid
DEG: Differentially expressed gene
DSP: Digital spatial profiling
HIE: Hypoxic ischemic encephalopathy
IFN: Interferon
IGF1: Insulin like growth factor 1
IRF8: Interferon regulatory factor 8
LPS: Lipopolysaccharide
MBP: Myelin basic protein
NLRP3: NLR family pyrin domain containing 3
OLIG2: Oligodendrocyte transcription factor 2
PSD95: Post synaptic density protein 95
PVL: Periventricular leukomalacia
ROI: Region of interest
SOX10: SRY box transcription factor 10
SPP1: Osteopontin
SUDI: Sudden unexpected death in infancy
