# Supplementary material for: Localized microglia dysregulation impairs central nervous system myelination in development
Source: Acta Neuropathol Commun. 2023 Mar 22;11:49. doi: 10.1186/s40478-023-01543-8 (PMC10035254; doi:10.1186/s40478-023-01543-8)
Supplement: Supplementary file 1 — Additional file 1: Table S1. Neuropathological and Clinical information of human infant brain samples used for digital spatial profiling and immunofluorescent assessment. Table S2. Clinical information of human infant cerebrospinal fluid samples used for SPP1 assessment. Fig. S1. Digital Spatial Transcriptomic profiling of human infant brain. A Overview images of ROI selection in injured infant cerebellum, stained for myelin (MBP; green), microglia/macrophages (CD68; red), and counterstained with Hoechst. B PCA plot of ROIs from female (orange) versus male (blue) human infant cerebellar white matter. C PCA plot of ROIs from human infant cerebellar white matter from cases with a neuropathological diagnosis of periventricular leukomalacia (PVL; purple) or hypoxic ischemic encephalopathy (HIE) or hypoxia (blue). Control infants are indicated in grey. D PCA plot of ROIs from human infant cerebellar white matter with age indicated. Individual infants indicated in different colours (injured are pink, control are green). E Volcano plot of control infants comparing those who died from Sudden Unexpected Death in Infancy (SUDI) versus other causes of death. Differentially expressed genes shown in grey, and interferon-related genes indicated in pink. Fig. S2. Oligodendrocyte lineage cell responses in human infant brain injury. A Mean proportion of IRF8+ cells that are OLIG2+ ± s.e.m. in cROI (n = 6 infants), iROI-M+ (n = 4 infants) and iROI-M− (n = 6 infants). B Mean proportion of IRF8+ cells that are CD68+ ± s.e.m. in cROI (n+6 infants), iROI-M+ (n = 4 infants) and iROI-M− (n = 6 infants).**P = 0.0039, Unpaired 2-tailed Student’s t-test. C Mean SOX10 normalized RNA counts ± s.e.m. for control and injured infants. Unpaired 2-tailed Student’s t-test, **P = 0.0080, n = 6 infants per group. D Mean OLIG2+ cells per mm2 ± s.e.m. in iROI-M+ and iROI-M−. n = 5 infants. Unpaired 2-tailed Student’s t-test, P > 0.05. E Mean OLIG2+ CC1- cells per mm2 ± s.e.m. in cROI (n = 6 infants), iRO [file 40478_2023_1543_MOESM1_ESM.docx]

Acta Neuropathologica Communications: Research

**Localized microglia dysregulation impairs central nervous system myelination in development**

**Supplementary Information:**

Supplemental Tables 1 and 2

Supplemental Figures and Legends S1-S3

Legends for Supplemental Excel Sheets 1-7

**Table S1. Neuropathological and Clinical information of human infant brain samples used for digital spatial profiling and immunofluorescent assessment**

| **Type** | **Number** | **Age at death** | **Sex** | **Neuropathological & clinical information** | **Methodology** |
| --- | --- | --- | --- | --- | --- |
| **Control infants** | 1 | 3d | F | Normal brain, SUDI | DSP & IHC |
|  | 2 | 5w | M | Normal brain, Septicaemia | DSP & IHC |
|  | 3 | 12d | F | Normal brain, cause of death N/A | DSP & IHC |
|  | 4 | 1d | F | Normal brain, intrapartum asphyxia | DSP & IHC |
|  | 5 | 1d | F | Normal brain, severe pulmonary hypoplasia, polycystic kidney disease | DSP & IHC |
|  | 6 | 5d | M | Normal brain, cause of death N/A | DSP & IHC |
|  | 7 | 4w | M | Normal brain, SUDI | IHC |
| **Injured infants** | 1 | 3d | F | HIE | DSP & IHC |
|  | 2 | 30 gw | M | HIE | DSP & IHC |
|  | 3 | 21d | F | PVL | DSP & IHC |
|  | 4 | 0d | F | Hypoxia | DSP & IHC |
|  | 5 | 1d | F | HIE | DSP & IHC |
|  | 6 | 8d | M | PVL | DSP & IHC |
|  | 7 | 16d | F | HIE | IHC |
|  | 8 | 4w | M | HIE | IHC |

D: days; w: weeks; gw: gestational weeks; M: male; F: female; SUDI: sudden unexpected death in infants; N/A: not available; HIE: hypoxic ischemic encephalopathy; PVL: periventricular leukomalacia; DSP: digital spatial transcriptomics; IHC: immunohistochemistry.

**Table S2. Clinical information of human infant cerebrospinal fluid samples used for SPP1 assessment**

| **Type** | **Case** | **Gestational age at birth**  **(weeks)** | **Age at lumbar puncture**  **(day of life)** | **Birth**  **weight**  **(g)** |
| --- | --- | --- | --- | --- |
| **Term** | 1 | 41.42 | 3 | 4082 |
|  | 2 | 41.14 | 20 | 3360 |
|  | 3 | 40.71 | 2 | 2770 |
|  | 4 | 40.14 | 2 | 3130 |
|  | 5 | 38 | 1 | 4730 |
|  | 6 | 37.85 | 2 | 3355 |
|  | 7 | 39.71 | 1 | 3410 |
|  | 8 | 36.85 | 2 | 3480 |
|  | 9 | 37.42 | 6 | 3410 |
|  | 10 | 41.71 | 2 | 4450 |
|  | 11 | 41.57 | 8 | 3505 |
|  | 12 | 40.57 | 6 | 3340 |
|  | 13 | 41.57 | 2 | 3840 |
|  | 14 | 37.42 | 1 | 3390 |
|  | 15 | 41.42 | 2 | 3840 |
|  | 16 | 41.42 | 1 | 2840 |
|  | 17 | 38.28 | 1 | 2212 |
|  | 18 | 40.71 | 2 | 3210 |
|  | 19 | 42 | 1 | 3390 |
|  | 20 | 37 | 2 | 3950 |
| **Preterm** | 1 | 25.28 | 24 | 827 |
|  | 2 | 29.28 | 9 | 1440 |
|  | 3 | 26 | 10 | 900 |
|  | 4 | 26.85 | 6 | 1310 |
|  | 5 | 25.28 | 11 | 825 |
|  | 6 | 27.85 | 4 | 1005 |
|  | 7 | 28.28 | 12 | 730 |
|  | 8 | 24.57 | 12 | 810 |
|  | 9 | 24.57 | 17 | 630 |
|  | 10 | 23.42 | 2 | 500 |
|  | 11 | 31.71 | 2 | 1520 |
|  | 12 | 27.57 | 28 | 1460 |
|  | 13 | 29 | 10 | 1240 |
|  | 14 | 25.71 | 28 | 935 |
|  | 15 | 28.28 | 27 | 1040 |
|  | 16 | 27.71 | 8 | 1100 |
|  | 17 | 29.42 | 5 | 1180 |

**Supplemental Figures and Legends**

**
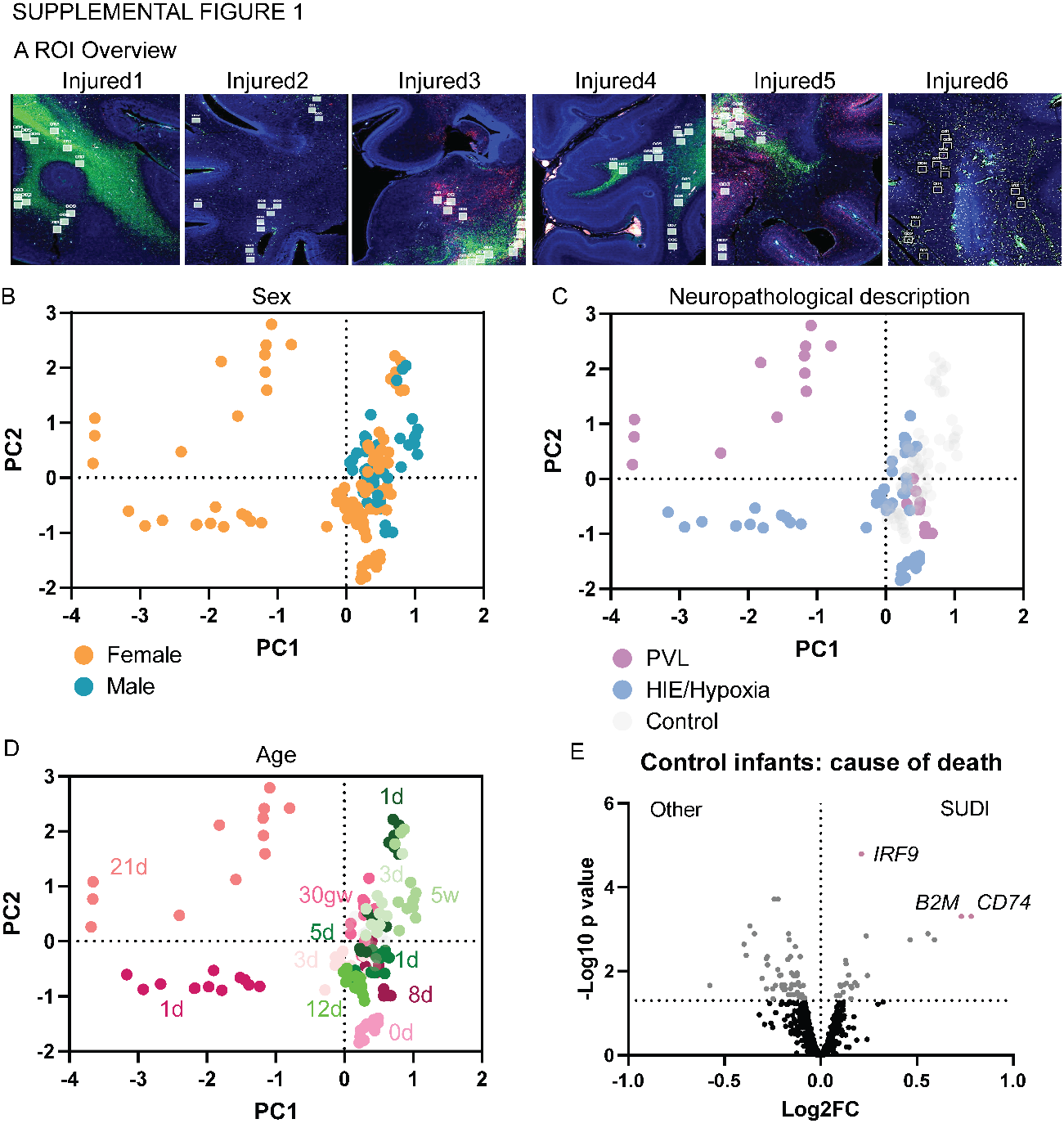
**

**Supplemental Fig. S1. Digital Spatial Transcriptomic profiling of human infant brain.**

1. Overview images of ROI selection in injured infant cerebellum, stained for myelin (MBP; green), microglia/macrophages (CD68; red), and counterstained with Hoechst.
2. PCA plot of ROIs from female (orange) vs male (blue) human infant cerebellar white matter.
3. PCA plot of ROIs from human infant cerebellar white matter from cases with a neuropathological diagnosis of periventricular leukomalacia (PVL; purple) or hypoxic ischemic encephalopathy (HIE) or hypoxia (blue). Control infants are indicated in grey.
4. PCA plot of ROIs from human infant cerebellar white matter with age indicated. Individual infants indicated in different colours (injured are pink, control are green).
5. Volcano plot of control infants comparing those who died from Sudden Unexpected Death in Infancy (SUDI) vs other causes of death. Differentially expressed genes shown in grey, and interferon-related genes indicated in pink.

**
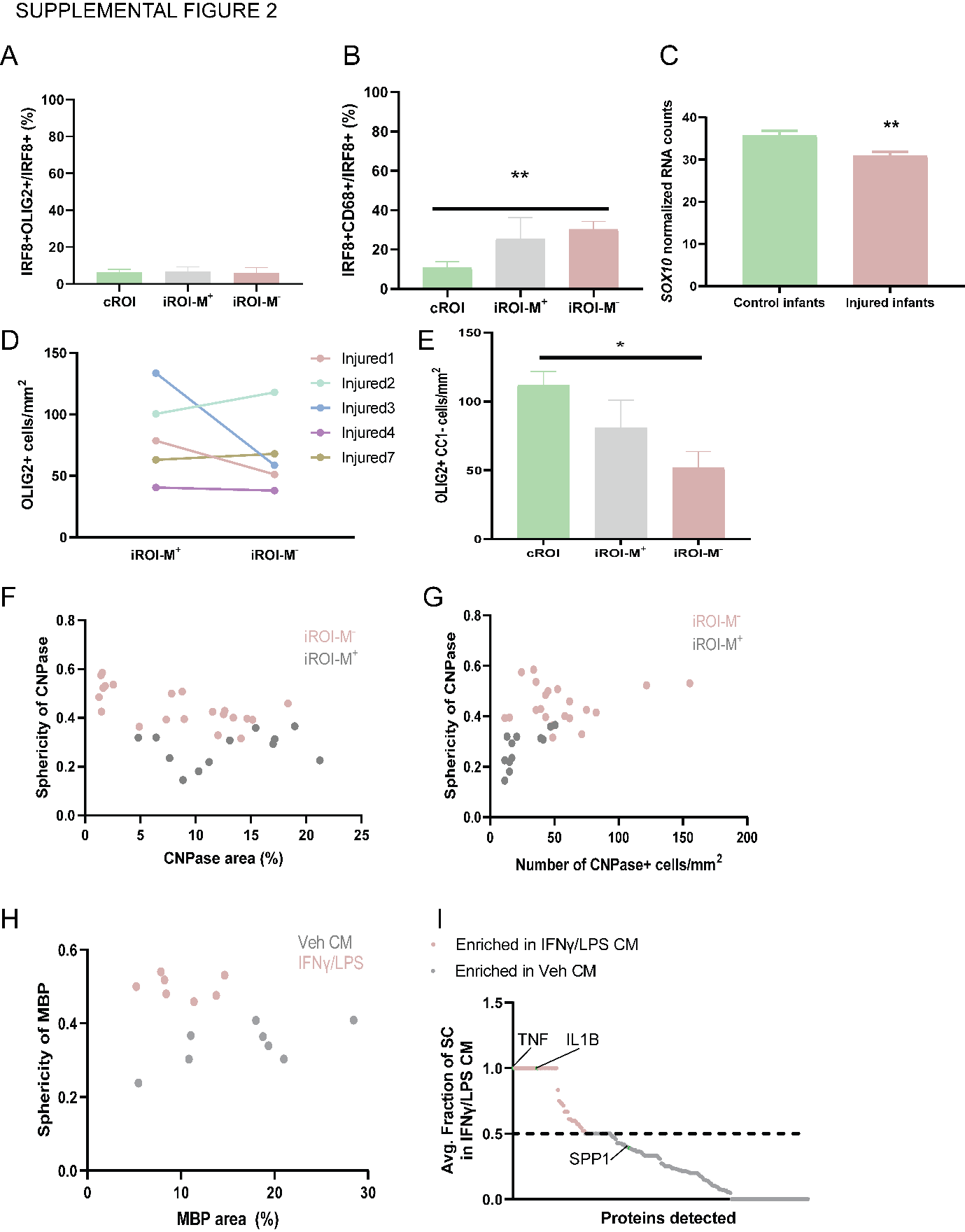
**

**Supplemental Fig. S2. Oligodendrocyte lineage cell responses in human infant brain injury.**

1. Mean proportion of IRF8+ cells that are OLIG2+ ± s.e.m. in cROI (n=6 infants), iROI-M^+^ (n=4 infants) and iROI-M^-^ (n=6 infants).
2. Mean proportion of IRF8+ cells that are CD68+ ± s.e.m. in cROI (n=6 infants), iROI-M^+^ (n=4 infants) and iROI-M^-^ (n=6 infants).**P=0.0039, Unpaired 2-tailed Student’s *t*-test.
3. Mean *SOX10* normalized RNA counts ± s.e.m. for control and injured infants. Unpaired 2-tailed Student’s *t*-test, **P=0.0080, n=6 infants per group.
4. Mean OLIG2+ cells per mm^2^ ± s.e.m. in iROI-M^+^ and iROI-M^-^. n=5 infants. Unpaired 2-tailed Student’s *t*-test, P>0.05.
5. Mean OLIG2+ CC1- cells per mm^2^ ± s.e.m. in cROI (n=6 infants), iROI-M^+^ (n=4 infants) and iROI-M^-^ (n=6 infants). *P=0.0108, Mann-Whitney test.
6. Sphericity (based on CNPase) versus percent CNPase area per respective ROI, in iROI-M^+^ (grey) and iROI-M^-^ (pink).
7. Sphericity (based on CNPase) versus number of CNPase+ cells per respective ROI, in iROI-M^+^ (grey) and iROI-M^-^ (pink).
8. Sphericity (based on MBP) versus MBP area in myelinating cultures exposed to conditioned media (CM) from microglia treated with PBS vehicle (grey) or IFNγ and LPS (pink).
9. Average fraction of total spectral counts (SC) in conditioned media (CM) from microglia treated with IFNγ and LPS, where proteins enriched in this condition are indicated in pink (e.g. TNF, IL1B) whereas proteins enriched in vehicle (Veh) control are indicated in grey (E.g. SPP1).

**
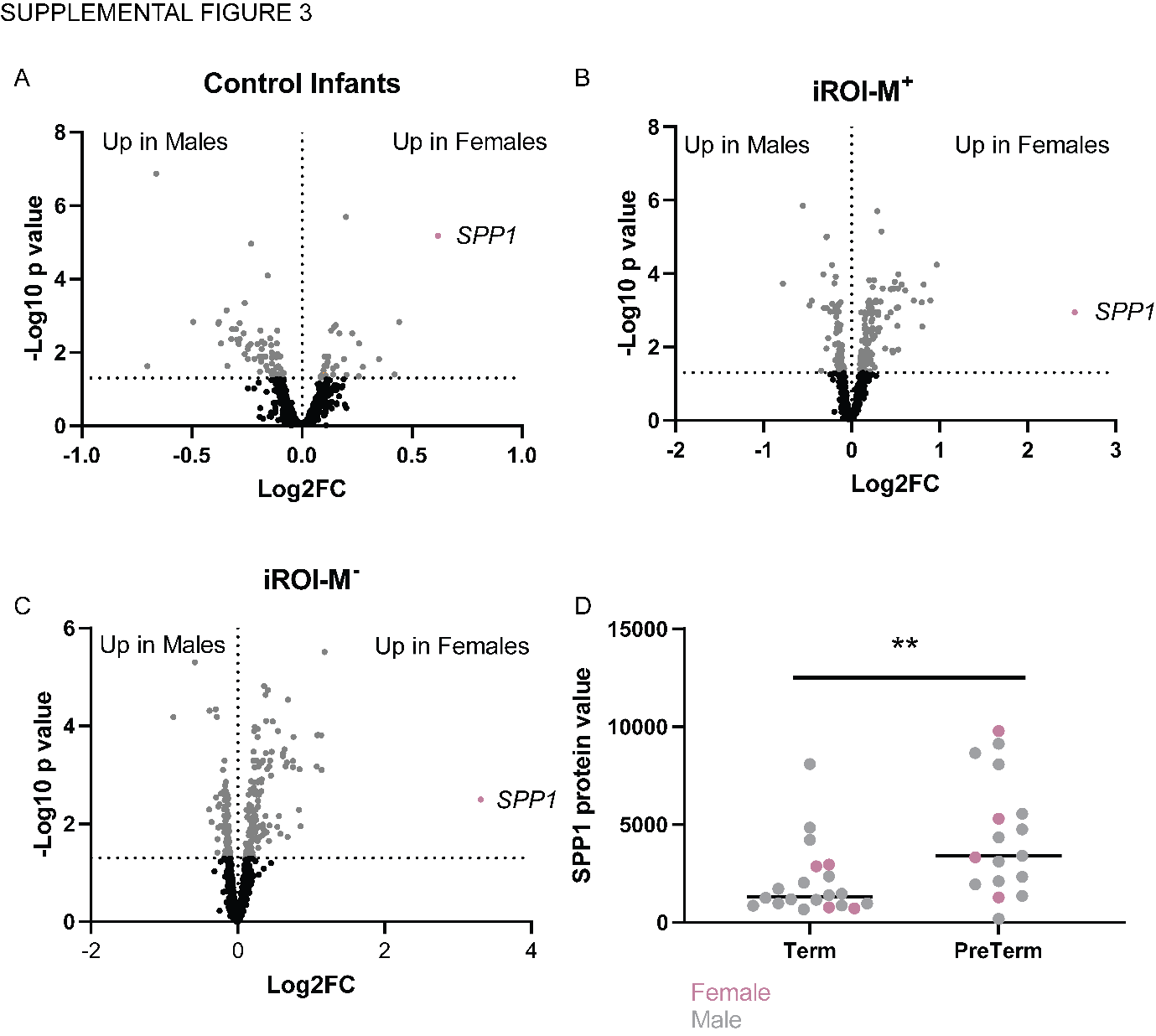
**

**Supplemental Fig. S3. Analysis of sex and Osteopontin regulation in human infant brain injury.**

1. Volcano plot of control infants, comparing female versus male gene expression. Differentially significantly regulated genes shown in grey. *SPP1* indicated in pink.
2. Volcano plot of iROI-M^+^, comparing female versus male gene expression. Differentially significantly regulated genes shown in grey. *SPP1* indicated in pink.
3. Volcano plot of iROI-M^-^, comparing female versus male gene expression. Differentially significantly regulated genes shown in grey. *SPP1* indicated in pink.
4. SPP1 protein levels in CSF of term vs preterm infants, with females indicated in pink and males indicated in grey. Mann-Whitney test, ***P*=0.0031, n=20 term infants and 17 pre-term infants.

**Supplemental Sheet Legends**

**Supplemental Sheet 1: Digital spatial transcriptomic profiling of human infant brain injury.**

Normalized RNA counts for 1825 targets, for 12 ROI per sample. N=6 control infants, 6 injured infants. ROI: region of interest. cROI: ROI from control infants. iROI-M^+^: ROI from normally myelinating regions of injured infants. iROI-M^-^: ROI from hypomyelinating regions of injured infants.

**Supplemental Sheet 2: Differentially expressed genes in respective iROI-M^-^ vs iROI-M^+^.**

Differentially expressed genes (DEG) in respective iROI-M^-^ vs iROI-M^+^ in 4 injured infants. FC: fold change.

**Supplemental Sheet 3: Pathway analysis of DEGs from respective iROI-M^-^ vs iROI-M^+^.**

Ingenuity Pathway Analysis (IPA) canonical pathways and predicted upstream regulators that are significantly regulated in comparison of respective iROI-M^-^ to iROI-M^+^ in 4 injured infants.

**Supplemental Sheet 4: Interferon-related DEGs from respective iROI-M^-^ vs iROI-M^+^.**

Lists of interferon-related DEGs in iROI-M^-^ vs iROI-M^+^ in 4 injured infants.

**Supplemental Sheet 5: Proteomic analysis of microglia conditioned media.** Average spectral counts and spectral count fraction following treatment with IFNγ/LPS or vehicle control. n=3 biological replicates per treatment condition.

**Supplemental Sheet 6: Differentially expressed genes from comparison of injured infants to control infants.**

Differentially expressed genes (DEGs) and interferon-related DEGs in injured vs control infants. N=6 control infants, 6 injured infants. FC: fold change.

**Supplemental Sheet 7: Differentially expressed genes from comparison of iROI-M^-^ to other ROI.**

Differentially expressed genes (DEGs) in all iROI-M^-^ vs all other ROI (cROI and iROI-M^+^ combined). N=6 infants with iROI-M^-^, 6 infants with cROI, and 4 infants with iROI-M^+^.
